# Impact of Mitochondrial A3243G Heteroplasmy on Mitochondrial Bioenergetics and Dynamics of Directly Reprogrammed MELAS Neurons

**DOI:** 10.3390/cells12010015

**Published:** 2022-12-21

**Authors:** Dar-Shong Lin, Yu-Wen Huang, Che-Sheng Ho, Tung-Sun Huang, Tsung-Han Lee, Tsu-Yen Wu, Zon-Darr Huang, Tuan-Jen Wang

**Affiliations:** 1 Department of Pediatrics, Mackay Memorial Hospital, Taipei 10449, Taiwan; 2 Department of Medicine, Mackay Medical College, New Taipei 25245, Taiwan; 3 Department of Medical Research, Mackay Memorial Hospital, Taipei 10449, Taiwan; 4 Department of Neurology, Mackay Children’s Hospital, Taipei 10449, Taiwan; 5 Department of Surgery, Mackay Memorial Hospital, Taipei 10449, Taiwan; 6 Department of Laboratory Medicine, Mackay Memorial Hospital, Taipei 10449, Taiwan

**Keywords:** induced neurons, mitochondrial diseases, MELAS, heteroplasmy, OXPHOS, bioenergetics, mitochondrial dynamics

## Abstract

The MELAS syndrome primarily affecting the CNS is mainly caused by the m.A3243G mutation. The heteroplasmy in different tissues affects the phenotypic spectrum, yet the impact of various levels of m.A3243G heteroplasmy on CNS remains elusive due to the lack of a proper neuronal model harboring m.A3243G mutation. We generated induced neurons (iNs) through the direct reprogramming of MELAS patients, with derived fibroblasts harboring high (>95%), intermediate (68%), and low (20%) m.A3243G mutation. iNs demonstrated neuronal morphology with neurite outgrowth, branching, and dendritic spines. The heteroplasmy and deficiency of respiratory chain complexes were retained in MELAS iNs. High heteroplasmy elicited the elevation in ROS levels and the disruption of mitochondrial membrane potential. Furthermore, high and intermediate heteroplasmy led to the impairment of mitochondrial bioenergetics and a change in mitochondrial dynamics toward the fission and fragmentation of mitochondria, with a reduction in mitochondrial networks. Moreover, iNs derived from aged individuals manifested with mitochondrial fission. These results help us in understanding the impact of various heteroplasmic levels on mitochondrial bioenergetics and mitochondrial dynamics in neurons as the underlying pathomechanism of neurological manifestations of MELAS syndrome. Furthermore, these findings provide targets for further pharmacological approaches of mitochondrial diseases and validate iNs as a reliable platform for studies in neuronal aspects of aging, neurodegenerative disorders, and mitochondrial diseases.

## 1. Introduction

Mitochondrial diseases are caused by mutations of either nuclear DNA (nDNA) or mitochondrial DNA (mtDNA). Of the mtDNA alternations, large-scale deletions and the point mutation of mtDNA lead to deficiencies in respiratory chain (RC) complexes impairing the activity of oxidative phosphorylation (OXPHOS) for ATP synthesis [1,2]. Nearly a half of OXPHOS defects resulting from the mitochondrial tRNA mutations manifest with a broad spectrum of clinical symptoms resulting from the varying involvement of multi-system impairment [1]. Mitochondrial encephalomyopathy, lactic acidosis, and stroke-like episodes (MELAS) syndrome is one of the most common OXPHOS diseases tending to develop in childhood and adulthood [3]. Although multiple organs are involved, the central nervous system is primarily affected in MELAS patients. Over 80% of cases of MELAS syndrome are caused by a common pathologic m.A3243G mutation in the mitochondrial tRNA^Leu^ (UUR) gene (MT-TL1) [3,4]. The pathogenic m.A3243G mutation causes amino acid misincorporation in the mitochondrial translation products and an assembly defect in complex I and IV, leading to the impairment of the OXPHOS function [5]. The level of heteroplasmy, defined as the proportion of wild-type (normal) and pathogenic mtDNA co-existing in the mutant cells, inversely correlates with age-of-onset and the phenotypic severity [6]. However, early studies in cybrids harboring nearly homoplasmic m.A3243G mutation showed variable extent of the translation defect and respiratory chain deficiency, suggesting that the cybrid models may not readily recapitulate the in vivo pathophysiology of MELAS syndrome [7,8,9,10].

Up to now, several cellular models have been used in unveiling the pathomechanism of MELAS syndrome. Nonetheless, studies related to the impact of various levels of m.A3243G heteroplasmy on neuronal aspect of MELAS syndrome have not been documented. In previous studies using the cytoplasmic hybrid technique, glioblastoma cybrid clones harboring 60% and 80% m.A3243G heteroplasmy showed a significant reduction in mitochondrial membrane potential (ΔΨm) and a decrease in ATP production, although the levels of reactive oxygen species (ROS) were compatible to that in wild-type controls [11]. Similar to these discrepant findings, neuroblastoma cybrids harboring 70% m.A3243G heteroplasmy showed a much higher expression of RC complexes I, II, and IV than that of wild-type controls and more fragmented mitochondria compared to cybrids harboring 100% m.A3243G heteroplasmy [12]. These findings in MELAS cybrid models suggest that cellular pathophysiology associated with m.A3243G heteroplasmy is altered by the tumorigenic background of cybrids. Alternatively, the development of human inducible pluripotent stem cell (iPSC) technology provides a patient-specific model for mitochondrial diseases. However, MELAS iPSC-derived neurons with 80% m.A3243G heteroplasmy showed an isolated deficiency of RC complex I, while the parental MELAS-iPSC had a normal amount of RC complexes I, III, and IV, concomitant with elevated RC complex II, in contrast to the parental MELAS-fibroblasts, which exhibited an isolated deficiency of RC complex IV [13]. These findings indicate that the pathophysiologic characteristics are altered during the reprogramming into iPSC and the differentiation into neurons. Furthermore, the mitochondrial rejuvenation and improvement of mitochondrial function during reprogramming into iPSC leads to differentiation into young neurons, which does not recapitulate the age-dependent pathophysiological characteristics of neurodegenerative diseases [14]. Recently, induced neurons (iNs) generated using direct reprogramming patient-derived fibroblasts have shown an advantage in maintaining the age-related signature and age-specific transcriptional profiles of parental cells [14]. Several studies have validated that iNs ensure a neuronal cellular modeling for the neurodegenerative diseases and mitochondrial disorders [15,16,17]. In a recent report, iNs harboring 75% m.A3243G heteroplasmy maintained the heteroplasmy load of the parental fibroblasts and showed significant low levels of bioenergetic profiles [18]. However, the impact of various levels of m.A3243G heteroplasmy upon the neurons of MELAS syndrome remained elusive. In the present study, we generated MELAS-derived iNs harboring high, intermediate, and low m.A3243G heteroplasmy. For the first time, the impact of various levels of heteroplasmy upon RC complexes, ROS levels, ΔΨm, mitochondrial bioenergetics, and mitochondrial dynamics in the neuronal aspect of MELAS syndrome was unveiled.

## 2. Material and Methods

### 2.1. Establish of Primary Skin Fibroblasts

Primary skin fibroblasts derived from skin biopsy of four individuals harboring m.A3243G mutations at different heteroplasmy and four age-/gender-compatible controls were established by obtaining informed consent in compliance with the Helsinki declaration and approval of the Institutional Review Board of MacKay Memorial Hospital. In brief, fibroblasts were maintained in standard Dulbecco’s modified Eagle medium (DMEM; Invitrogen, Grand Island, NY, USA), supplemented with 10% (*v/v*) fetal bovine serum (FBS; Gibco, Grand Island, NY, USA) and 1% penicillin G/streptomycin sulfate at 37 °C, in a 5% (*v/v*) CO_2_ humidified incubator.

### 2.2. Measurement of Mutant Heteroplasmy

Genomic DNA used in quantitative PCR (qPCR) reactions was extracted from cells using commercial QIAamp DNA Blood Mini Kit (Qiagen, Germantown, MD, USA) according to the manufacturer’s instruction. The levels of heteroplasmy in mitochondrial DNA A3243G mutation were measured using allele refractory mutation system (ARMS)-based qPCR analysis according to previously described method [19]. In brief, each qPCR reaction contains an aliquot of DNA sample, ARMS primers, and FastStart Essential DNA Green Master (Roche life science, Mannheim, Germany) for PCR amplification using the LightCycler^®^ *96 Instrument* (Roche life science). The delta (∆) cycle threshold (Ct) value was calculated as (ΔC_T_ = CT^wildtype^ − CT^mutant^). The level of m.A3243G heteroplasmy (%) was calculated using formula: 1/[1 + (1/2) ^ΔCT^] × 100%.

### 2.3. Direct Reprogramming of Primary Skin Fibroblasts to iNs

Primary skin fibroblasts were converted to induced neurons (iNs) directly using slight modifications to methods described previously [20,21]. Controls and MELAS patient-derived fibroblasts were seeded at a density of 1 × 10^4^ cells/cm^2^ on matrigel (BD bioscience, Erembodegem, Belgium)-coated culture plates. The following day, fibroblasts were transduced with lentiviral vectors containing shRNA against human PTBP1 (polypyrimidine tract-binding protein 1), selected with 1 mg/mL puromycin, and replaced with N3 medium (Gibco) supplemented with neuronal induction factors as previously described for 3 days [20]. The day after, the cells were switched into N3 medium supplemented with 2% FBS and after a further three days, the medium was replaced with DMEM/F12 and Neurobasal medium supplemented with neural growth factors and small molecules CHIR99021, forskolin, forsomorphin, ascorbic acid, and valproic acid (Sigma-Aldrich, St. Louis, MO, USA) for a further 3 days as previously described [21]. Neuronal cells were identified through the staining of antibody against Tuj1 (Abcam, Boston, MA, USA) and phalloidin reagent (Abcam). Neuronal purity was calculated as the ratio of total number of Tuj1-positive cells to the total cells indicated using nuclear staining.

### 2.4. Western Blotting

Cells were homogenized and lysed in T-PER™ Tissue Protein Extraction Reagent (Thermo Fisher Scientific, Waltham, MA, USA) supplimented with Halt^TM^ protease inhibitor cocktail (Thermo Fisher Scientific). Supernatant was collected after centrifugation and quantified using the BCA protein assay (Thermo Fisher Scientific) according to the manufacturer’s instructions. Protein samples were denatured, resolved through 10% sodium dodecyl sulfate polyacrylamide gel electrophoresis, and transferred to PVDF membrane (Millipore, Bellerica, MA, USA). The membrane was blocked in TBST [20 mMf Tris-HCl (pH 7.5), 150 mM NaCl, 0.1% Tween 20] buffer with 0.5% nonfat milk, and incubated with primary antibodies against subunit of respiratory chain complex I (NDUFB8; Abcam), II (SDHB; Abcam), III (UQCR2; Abcam), IV (MTCO2; Abcam), V (ATP5AA), and porin (Abcam), overnight at 4 °C. After washes with TBST buffer, the membrane was incubated with horseradish peroxidase (HRP)-conjugated secondary antibody, washed, and visualized using Immobilon Western Chemiluminescent HRP Substrate (Millipore).

### 2.5. Detection of Mitochondrial Membrane Potential (ΔΨm)

iNs were cultured in 6-well 35-mm plates, followed by incubation with mitochondrial potential dye, 100 nM TMRE (tetramethyl rhodamine methyl ester; Mitochondrial Membrane Potential Assay kit ab113852; abcam) in pre-warmed DMEM medium, for 30 min at 37 °C in 5% CO_2_ incubator in the dark, counterstained with Hoechst 33342 (Sigma-Aldrich) (1 μL/mL) for 2 min and washed with 0.2% FBS in HBSS. The fluorescent intensity and image were measured with ImageXpress Micro 4 (Molecular Devices, San Jose, CA, USA), and the mean intensity of TMRE fluorescence per cell was quantified for statistical analysis. All measurements, normalized for number of cells, were presented as mean ± SD.

### 2.6. Measurement of Mitochondrial Reactive Oxygen Species

Production of ROS in iNs was visualized through staining with fluorescent dye MitoSox Red (Thermo Fisher Scientific) reagent. To prevent the adverse effects of MitoSOX on mitochondrial membrane potential and mitochondrial function [22,23,24], iNs were incubated with a submicromolar concentration of 1 μM MitoSox Red for 10 min at 37 °C in the dark, briefly washed with 0.2% FBS in HHBS to remove the excess dye, counterstained with Hoechst 33342 (Sigma-Aldrich) (1 μL/mL) for 2 min, and washed with 0.2% FBS in HBSS. The fluorescent intensity was read with a microplate reader (Infinite M200PRO, TECAN, Mannedorf, Switzerland). The mean fluorescence intensity per cell was quantified. All measurements, normalized for number of cells, were presented as mean ± SD.

### 2.7. Cellular Bioenergetics

Induced neurons through transduction of fibroblasts with lentiviral vectors containing shRNA against human PTBP1 were seeded at a density of 2 × 10^4^ cells/well in Seahorse XF 24-well plate, followed by administration of small molecules for neuronal maturation as described above, and assessed on an XF24 Analyzer (Seahorse Biosciences, North Billerica, MA, USA) for measurement of mitochondrial bioenergetics. Oxygen consumption rate (OCR) of cells was recorded in real time at baseline and after sequential additions of 1 mM oligomycin, 1 mM FCCP (Carbonyl-cyanide-p-trifluoromethoxy-phenyl-hydrazone), and 0.4 mm com-bination of antimycin A/rotenone, respectively, as previously described [25]. OCR was used as an indicator of aerobic oxidation of glucose. The results of OCR were normalized with protein content and expressed as pmol/min/mg protein.

### 2.8. Mitochondria Morphology and Network

Mitochondria morphology and network were analyzed using images processing with ImageJ plugin toolset Mitochondrial Analyzer [26]. iNs were stained with MitoTracker^®^ Red CMXRos (Thermo Fisher Scientific) according to manufacturer’s instruction. Images were captured under confocal microscope. Images of iNs after removal of background and noise were segmented, thresholded, and binarized to produce a morphological skeleton for qualification of mitochondrial morphology and network. After this, Mitochondrial Analyzer generates parameters (area, mean perimeter, aspect ratio, form factor) to quantitatively describe the mitochondrial morphology, as well as parameters (branches, total branch length, mean branch length, branch endpoints) related to the network complexity of mitochondria [26]. The aspect ratio is defined as the ratio of centerline long axis and short axis. A minimum aspect ratio of 1 reflects a perfect circle, and an increase in aspect ratio reflects a more elongated and elliptical mitochondrion [26]. The form factor is calculated as (4π × Area/perimeter) and defined as the inverse value of circularity to describe the mitochondria’s shape. Form factor is 1 for a circular and unbranched mitochondrion, and form factor increases as mitochondria elongate and develop more branches [26]. All measurements were presented as mean ± SD.

### 2.9. Statistical Analysis

All measurements were obtained from at least three independent experiments and results were expressed as the mean ± SD. For parametric data, one-way ANOVA, two-way ANOVA or Student’s *t* test was used for comparing between two groups of values statistically. *p*-values less than 0.05 were considered significant.

## 3. Results

### 3.1. Generation of iNs

**Figure 1:**
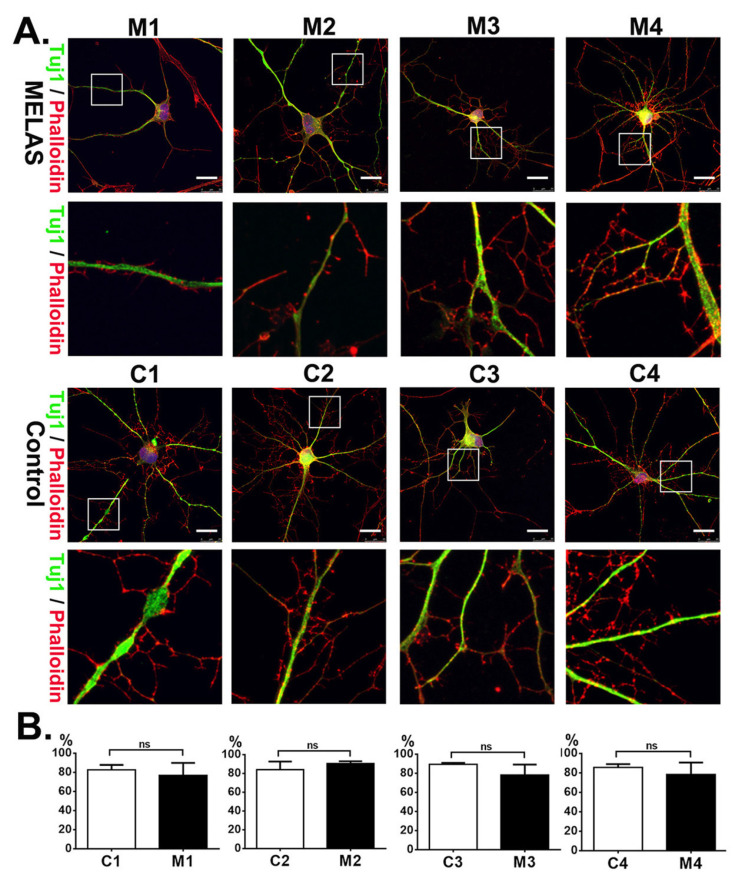
Induced neurons (iNs) generated through direct reprogramming of MELAS and control fibroblasts. (**A**) MELAS (M1, M2, M3, M4) and control (C1, C2, C3, C4) iNs immunostained for Tuj1 and F-actin (phylloidin) reveal neuronal morphology with the neuronal soma, elaborate neurites, and dendritic spines. The neurite and dendritic spines in the squared box are shown in high magnification in under panel. (**B**) Neuronal purity of iNs is calculated and plotted. The results are expressed as the mean ± SD of three independent experiments. Statistical significance was calculated using Student’s *t* test. ns: not significant. Scale bar, 25 μm.

Primary skin fibroblast derived from three MELAS syndrome patients (M1, 7-year-old male; M2, 17-year-old male; M3, 19-year-old male), one asymptomatic female carrier (M4, 56-year-old female), and four age-/gender-matched controls (C1, 7-year-old male; C2, 15-year-old male; C3, 19-year-old male; C4, 50-year-old female) were used for direct reprogramming into iNs. Fibroblasts were transinfected with lentiviral vectors encoding shRNA against PTBP1, which is the RNA-binding protein repressing the neuronal alternative splicing during the embryonic CNS development, to drive cells into the neuronal pathway [20,27]. After transduction, induced neuronal cells were treated with small molecules to facilitate neuronal maturation [21]. Twelve days after induction, cells showed neuronal morphology and expressed immunoreactivity against neuronal marker Tuj-1. Furthermore, the iNs were stained with phalloidin to demonstrate the filamentous actin (F-actin) in the cell bodies, axons, and dendrites. (Figure 1A). Overall, the iNs showed a mature neuronal morphology with outgrowth and branching neuritis with dendritic spines.

The neuronal purity was defined based on the ratio of Tuj-1 positive cells over the total reprogrammed cells. The neuronal purity of MELAS iNs M1, M2, M3, and M4 was 76.8%, 90.5%, 78.2% and 78.5%, respectively. The neuronal purity of control iNs C1, C2, C3, and C4 was 82.6%, 84%, 89.5%, and 85.6%, respectively. There was no significant difference between the MELAS iNs and control iNs (Figure 1B).

### 3.2. iNs Retained Stable m.A3243G Heteroplasmy

**Figure 2:**
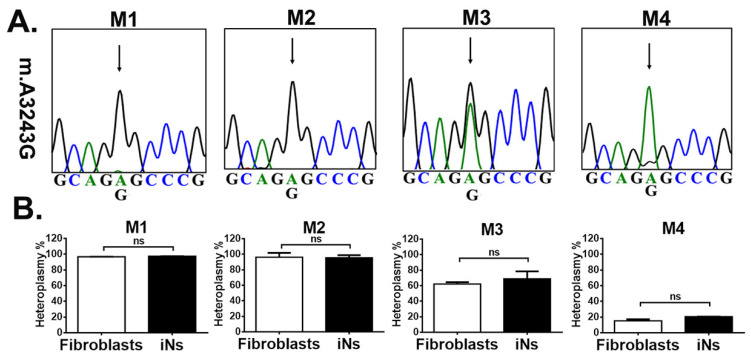
Heteroplasmy of MELAS fibroblasts and MELAS iNs. (**A**) Sequencing chromatogram shows the heteroplasmic m.A3243G mutation in fibroblasts derived from MELAS patients (M1, M2, M3, M4). (**B**) Heteroplasmy levels of m.A3243G mutation quantified through qPCR assay in MELAS fibroblasts and MELAS iNs derived from MELAS fibroblasts. Results are expressed as mean ± SD of three independent experiments. Statistical significance was calculated using Student’s *t* test. ns: not significant.

We examined whether m.A3243G heteroplasmy changes with direct reprogramming. The m.3243G mutation was sequenced (Figure 2A) and levels of heteroplasmy (Figure 2B) were measured in three passages of MELAS-derived fibroblasts and iNs, respectively, with ARMS-based quantitative PCR method. The average level of heteroplasmy in the three passages of M1-to-M4-derived fibroblasts was 97%, 96%, 62%, and 15%, respectively (Figure 2B). In corresponding with its parental fibroblasts, the average level of heteroplasmy in iNs through the direct reprogramming of M1 to M4 fibroblasts was 97%, 95%, 68%, and 20%, respectively (Figure 2B). Intriguingly, there was no significant difference in levels of heteroplasmy between MELAS-derived fibroblasts and its corresponding iNs. This result indicated that the heteroplasmy of the m.A3243G mutation retained stable through direct reprogramming.

### 3.3. RC Complexes Deficiency in MELAS iNs

**Figure 3:**
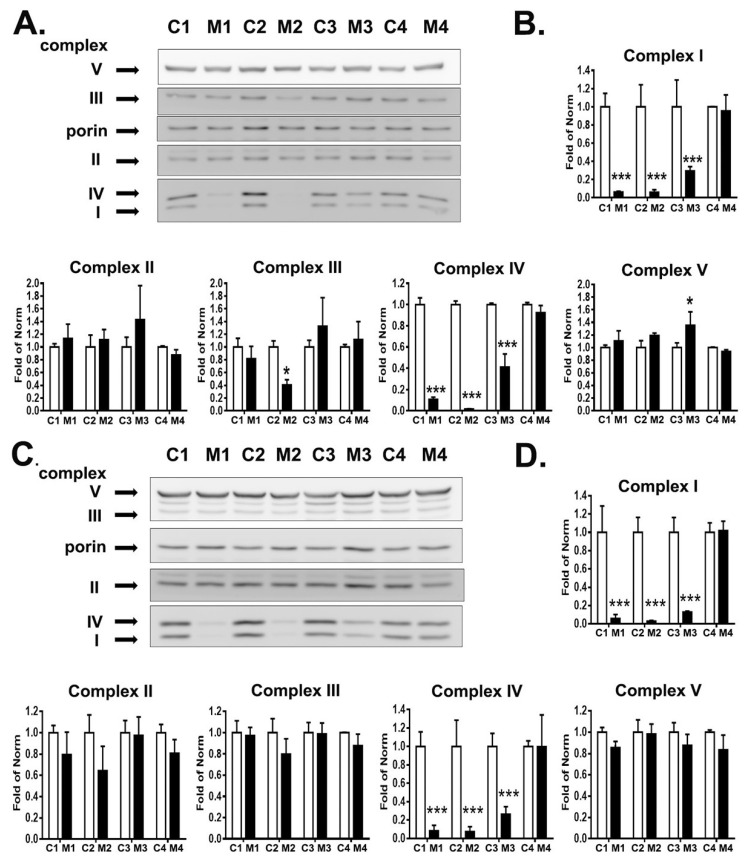
Expression of respiratory chain complexes in MELAS fibroblasts and MELAS iNs. (**A**) Western blotting analysis of respiratory chain complexes I–V in fibroblasts derived from controls (C1, C2, C3, C4) and MELAS (M1, M2, M3, M4) patients. (**B**) Quantification of respiratory complexes in fibroblasts after normalization to porin. Results are expressed as mean ± SD of three independent experiments. (**C**) Western blotting analysis of respiratory chain complexes I–V in iNs derived from control (C1, C2, C3, C4) and MELAS (M1, M2, M3, M4) fibroblasts. (**D**) Quantification of respiratory chain complexes in iNs after normalization to porin. Results are expressed as mean ± SD of three independent experiments. Statistical significance was calculated through two-way ANOVA. * *p* < 0.05 and *** *p* < 0.001 vs. control.

Mitochondrial DNA A3243G mutation causes amino acid misincorporation in translation, leading to RC assembly defects and deficiency of complexes I and IV [5,28]. In accord with this notion, we analyzed the RC complexes in both MELAS fibroblasts and iNs through western blotting (Figure 3A,C). As anticipated, the fibroblasts of M1 and M2 harboring high heteroplasmy showed profound deficiency of complexes I (6% and 6% of norm, respectively) and IV (10% and 2% of norm, respectively), and M3 fibroblasts with intermediate heteroplasmy showed moderate deficiency of complexes I (30% of norm) and IV (40% of norm), while levels of RC complexes of M4 fibroblasts with low heteroplasmy were compatible with that of normal controls (Figure 3B). Furthermore, M2 fibroblasts showed deficiency of complex III (40% of norm) and M3 fibroblasts showed upregulation of complex V (135% of norm). In line with the findings in fibroblasts, M1 and M2 iNs presented with profound deficiency of complexes I (6% and 3% of norm, respectively) and IV (8% and 7% of norm, respectively), similar to that noted in its parental fibroblasts (Figure 3D). M3 iNs showed more obvious deficiency of complexes I (13% of norm) and IV (26% of norm) than that observed in parental M3 fibroblasts. The levels of RC complexes in M4 iNs were indistinguishable with that of the controls. These findings indicated the tissue-specific vulnerability to mitochondrial heteroplasmic mutations.

### 3.4. High Heteroplasmy Increased ROS Production

**Figure 4:**
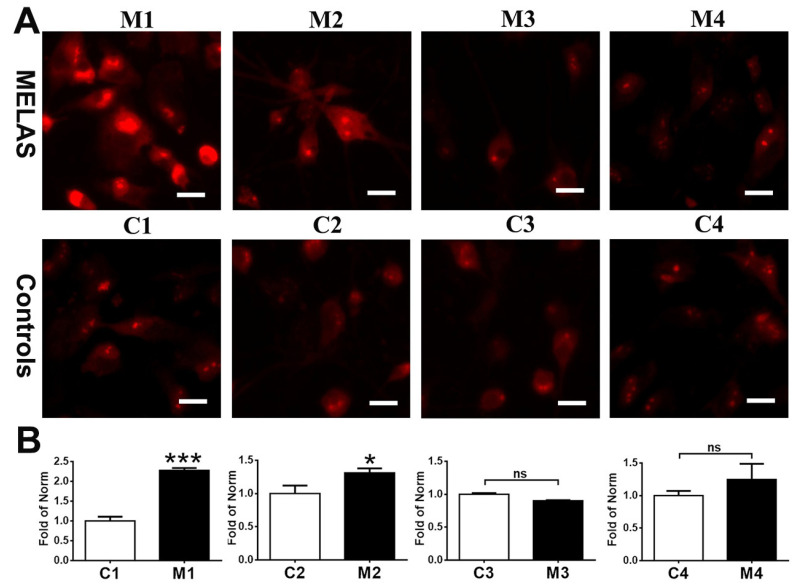
Elevation in mitochondrial superoxide in MELAS iNs. (**A**) A representative image of iNs staining with MitoSox Red for detection of mitochondrial superoxide. (**B**) Quantification of levels of mitochondrial superoxide in control (C1, C2, C3, C4) and MELAS (M1, M2, M3, M4) iNs. Results are expressed as mean ± SD of three independent experiments. Statistical significance was calculated using Student’s *t* test. * *p* < 0.05 and *** *p* < 0.001 vs. control. ns: not significant. Scale bar, 20 μm.

Deficiency of RC complexes impairs the mitochondrial function leading to the overproduction of ROS in the form of superoxide anion or hydrogen peroxide, resulting in cellular dysfunctions and initiation of apoptosis, senescence, and cell death in CNS. To understand the impact of m.A3243G heteroplasmy on the production of ROS, MELAS iNs were stained with MitoSOX Red (Figure 4A). It was noted that M1 and M2 iNs, both with high levels of m.A3243G mutation heteroplasmy, had excessive levels of superoxide compared with that of control iNs (Figure 4B). M3 iNs with intermediate heteroplasmy had a mildly reduced level of superoxide and M4 iNs with low heteroplasmy had levels of superoxide compatible with that of the control iNs.

### 3.5. Reduction in ΔΨm in iNs with High Heteroplasmy

**Figure 5:**
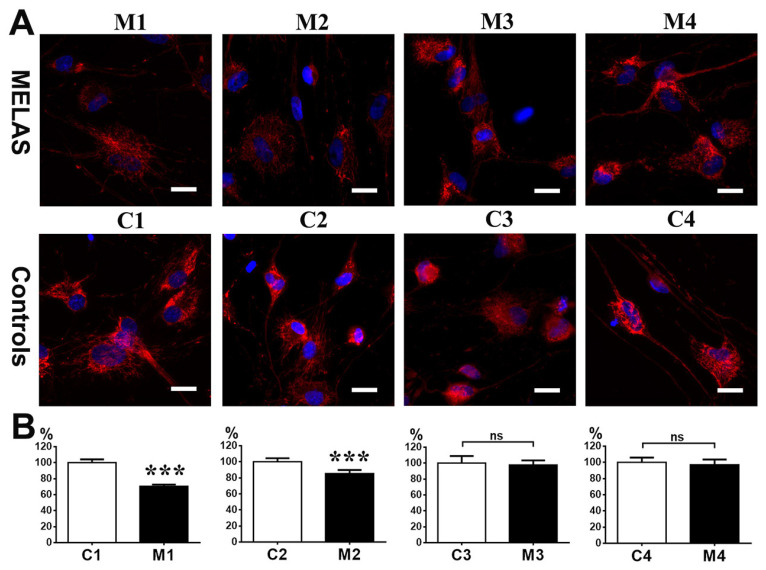
Disruption of mitochondrial membrane potential in MELAS iNs. (**A**) A representative image of iNs staining with TMRE for detection of mitochondrial membrane potential. Cells were counterstained with Hoechst. (**B**) Quantification of levels of mitochondrial membrane potential in controls (C1, C2, C3, C4) and MELAS (M1, M2, M3, M4) iNs. Results are expressed as mean ± SD of three independent experiments. Statistical significance was calculated using Student’s *t* test. *** *p* < 0.001 vs. control. ns: not significant. Scale bar, 20 μm.

Depolarization of mitochondrial membrane potential is frequently associated with increase in ROS in the pathogenesis of mitochondrial diseases. Accordingly, we measured the ΔΨm through staining iNs with TMRE (Figure 5A), which is a voltage-sensitive fluorescent dye accumulating in mitochondria evenly. As expected, and in accord with the findings of excessive production of ROS, both M1 and M2 iNs showed a significant reduction in ΔΨm compared to that of the control iNs (Figure 5B). The ΔΨm of both M3 and M4 iNs was indistinguishable from that of the control iNs. Taken together, high m.A3243G heteroplasmy induced excessive ROS production and depolarization of ΔΨm in MELAS iNs, while MELAS iNs with intermediate and low heteroplasmy was able to maintain homeostasis of ROS production and ΔΨm.

### 3.6. Impact of Heteroplasmy on the Bioenergetics

**Figure 6:**
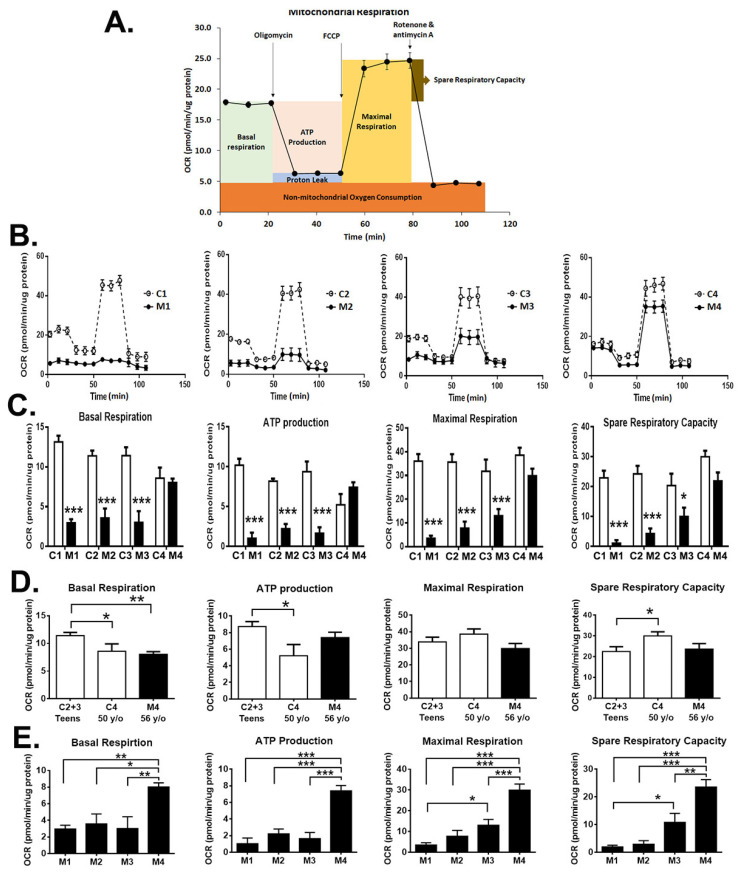
Bioenergetics profiles of MELAS iNs. (**A**) General scheme depicting oxygen consumption rate (OCR) following sequential addition of oligomycin (ATP synthase inhibitor), FCCP (uncoupler), and Rotenone + Antimycin A (electron transport chain inhibitors) in cells to derive parameters of mitochondrial respiration. (**B**) Representative profile of bioenergetic analysis in control (C1, C2, C3, C4) and MELAS (M1, M2, M3, M4) iNs measured with a Seahorse XF-24 flux analyzer. (**C**) Parameters of bioenergetic profiles including basal respiration, ATP production respiration, maximal respiration, and spare respiration capacity are plotted. Results are expressed as mean ± SEM of three independent experiments: * *p* < 0.05 and *** *p* < 0.001 vs. control. (**D**) comparison of mitochondrial bioenergetics between teens control (C2 + C3), aged control C3, and aged M4 iNs. (**E**) comparison of mitochondrial energetics between MELAS iNs. Results are expressed as mean ± SEM. Statistical significance was calculated using two-way ANOVA (**C**), and one-way ANOVA (**D**,**E**): * *p* < 0.05, ** *p* < 0.01, and *** *p* < 0.001.

To determine the impact of m.A3243G heteroplasmy on mitochondrial respiration activity, the OCR in the iNs was monitored in real time using the Seahorse XF Analyzer. Bioenergetic assessment was determined through monitoring the OCR after serial addition of the RC complexes inhibitors (Figure 6A). The oxygen consumption due to basal respiration, ATP-production, maximal respiration, and spare respiratory capacity was determined accordingly (Figure 6B).

In the assessment of oxygen consumption at basal condition (basal respiration), M1 and M2 iNs showed 78% and 69% reduction in OCR, respectively, and M3 iNs showed 74% reduction in OCR compared to the controls (Figure 6C). The M4 iNs maintained the basal respiration compatible with the controls (Figure 6C).

Subsequential addition of complex V inhibitor oligomycin allowed the measurement of oxygen consumption used for synthesizing ATP (ATP production respiration) in cells. The M1, M2, and M3 iNs showed 90%, 73%, and 82% reduction in OCR, respectively, in ATP production respiration compared to controls, while that of M4 iNs was in accord with the controls (Figure 6C).

The maximal uncoupled respiration (maximal respiration) of the mitochondrial electron transport process for energy generation was estimated through promoting maximal OCR from additions of chemical uncoupling agents FCCP preceded by the inhibition of oxidative phosphorylation using oligomycin (Figure 6C). The results demonstrated that the reduction in maximal respiration was in line with the levels of heteroplasmy in iNs. The M1 iNs had the most pronounced decrease (91.2% reduction) of maximal respiration, followed by the M2 iNs with a 78.2% decrease and the M3 iNs with a 59% decrease in OCR with respect to the corresponding control, while the M4 iNs had a moderate lower level of maximal respiration, but without significant difference, than that of the control (Figure 6C).

The difference between the maximal and basal respiration is interpreted as a measurement of the spare respiratory capacity. In line with the measurement of maximal respiration, the spare respiratory capacity in MELAS iNs was remarkably reduced in the M1 with a 95.5% decrease, followed by the M2 cells with an 82% decrease, and the M3 cells with a 51% decrease in OCR as respect to the corresponding control (Figure 6C). Consistently, the M4 iNs showed a moderately reduced level of spare respiration, albeit without a significant difference in comparison with that of the control (Figure 6C). Overall, the MELAS iNs harboring high (>95%) and intermediate (68%) m.A3243G heteroplasmy showed an impairment of the mitochondrial function. While the MELAS iNs harboring low m.A3243G heteroplasmy maintained the mitochondrial respiration compatible with that of the control.

Furthermore, the mitochondrial function was compared between the teen controls (C2 and C3), aged control (C4), and aged MELAS M4 iNs (Figure 6D). The aged C4 iNs showed a significantly moderate reduction in basal respiration and ATP production, although an increase in spare respiratory capacity, in respect to the teen controls (C2 and C3). Similarly, the aged M4 iNs showed a significant decrease in basal respiration in respect to the teenager controls. This result indicated the decreased mitochondrial function in neurons derived from aged individuals in comparison to young individuals. While the ATP production and the maximal respiration of the aged M4 iNs were moderately reduced without significant difference in respect to the teen controls, suggesting an adaptive regulation using mitochondrial-nuclear communication to maintain homeostasis during aging [29].

Additionally, we compared the mitochondrial function between the MELAS iNs. It showed that the M4 iNs harboring low m.A3243G heteroplasmy had significantly better mitochondrial function compared to that of the iNs harboring high (M1 and M2) and intermediate (M3) heteroplasmy (Figure 6E). This result was parallel with the activity of the RC complexes in the MELAS iNs.

### 3.7. Impact of Heteroplasmy on Mitochondria Dynamics

**Figure 7:**
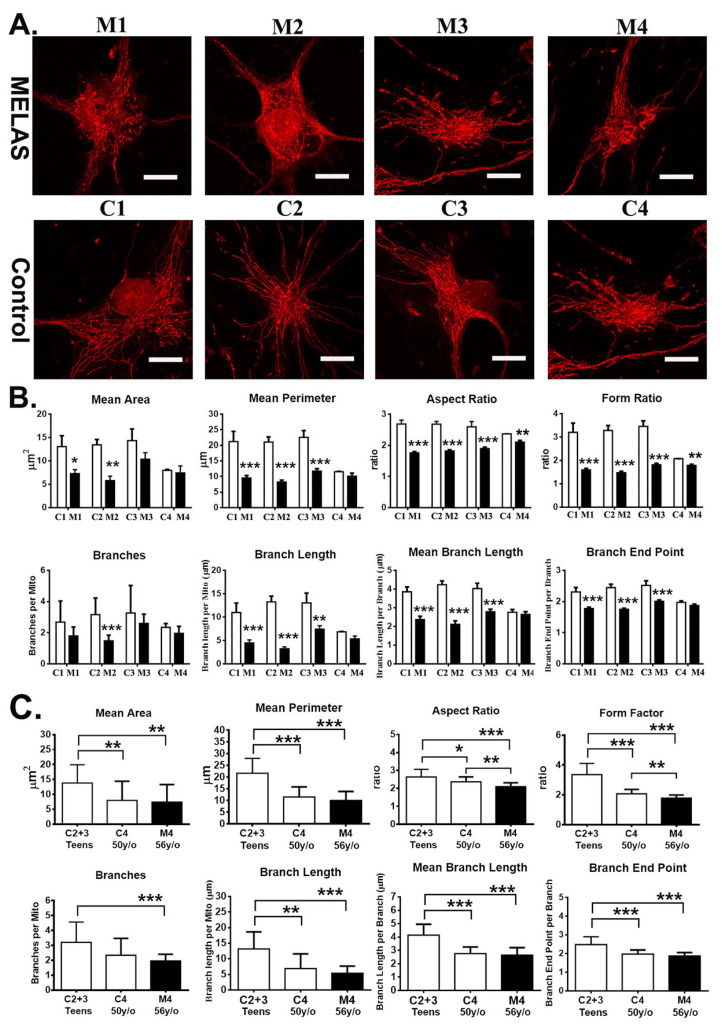
Alternation of mitochondrial dynamics in MELAS iNs. (**A**) Representative image of mitochondrial morphology in iNs staining with MitoTracker^®^ Red CMXRos. (**B**) Quantitative analysis of mitochondrial morphology and network parameters in control (C1, C2, C3, C4; *n* = 11~14 neurons each group) and MELAS (M1, M2, M3, M4; *n* = 12~15 neurons each group) iNs. Results are expressed as mean ± SD of three independent experiments: * *p* < 0.05, ** *p* < 0.01, and *** *p* < 0.001 vs. control. (**C**) comparison of mitochondrial morphology between teen controls (C2 + C3), aged control C3, and aged M4 iNs. Results are expressed as mean **+** SD of three independent experiments. Statistical significance was calculated using two-way ANOVA (B), and one-way ANOVA (**C**): * *p* < 0.05, ** *p* < 0.01, and *** *p* < 0.001. Scale bar, 20 μm.

To understand the impact of m.A3243G heteroplasmy on the mitochondrial morphology in the iNs, the mitochondria were labelled with the MitoTrackerRed (Figure 7A), visualized under a confocal microscopy, and analyzed quantitatively using the ImageJ plugin Mitochondrial Analyzer. The morphological characteristics of mitochondria size were defined by area and perimeter, and the shape of the mitochondria was defined by the form factor and aspect ratio [26]. The smaller values of area, perimeter, aspect ratio, and form factor indicate the mitochondria dynamics shifting toward fission and fragmentation. Herein, the M1 and M2 iNs harboring high heteroplasmy showed a remarkable and significant reduction in mitochondrial area, perimeter, form factor, and aspect ratio in respect with the controls (Figure 7B). Similarly, the M3 iNs harboring intermediate heteroplasmy had a significant reduction in mitochondrial perimeter, form factor, and aspect ratio in comparison with the control, and the average area of the mitochondrion were smaller, but without significant difference, as respect to the control (Figure 7B). The M4 iNs harboring low heteroplasmy had a mitochondrial size indistinguishable from that of the control iNs, albeit showing a reduction in mitochondrial shape in respect to the control. Of note, both the M4 iNs and C4 iNs were derived from the aged individuals and showed a significant reduction in mitochondrial area, perimeter, form factor, and aspect ratio in respect to the C2 and C3 iNs derived from the teen individuals (Figure 7B). Overall, the MELAS iNs harboring high and intermediate heteroplasmy showed mitochondrial fission and fragmentation in comparison to the filamentous mitochondria of the controls.

Furthermore, the network complexity of mitochondria was described quantitatively by analyzing the number of branches, total branch length, mean branch length, and number of branch end points [26]. The results showed a significant decrease in mitochondrial branches in the M2 iNs compared to that of the control, and that of the M1, M3, and M4 iNs were decreased but without reaching a significant difference in respect to the controls (Figure 7B). Of note, the mitochondrial network parameters of the total branch length, mean branch length, and the amount of branch end points were significantly reduced in the M1, M2, and M3 iNs in respect to the controls. The M4 iNs had mitochondrial network parameters compatible with that of the control. Overall, the MELAS iNs harboring high and intermediate heteroplasmy had a significant reduction in the complexity of the mitochondrial network.

Furthermore, mitochondrial morphology and network complexity were compared between the teen controls (C2 and C3), aged control (C4), and the aged MELAS M4 iNs (Figure 7C). It showed that the parameters related to mitochondrial morphology and network complexity of the C4 and M4 iNs were significantly reduced in respect to the teen controls (Figure 7C). This finding is in line with the findings of previous studies in that the iNs derived from the aged donor fibroblasts display a variety of mitochondrial aging phenotypes [30].

## 4. Discussion

Herein, we used the state-of-the-art iNs generated through the direct reprogramming of somatic cells technology to provide a neuronal model of MELAS syndrome. The MELAS iNs exhibited the morphology of matured neurons and retained the levels of heteroplasmy and the expression of RC complexes. Specifically, we report the in-depth phenotyping of mitochondrial function and mitochondrial morphology in theMELAS iNs harboring various levels of heteroplasmy to unveil the hitherto unknown impact of heteroplasmy on the neuronal phenotype of MELAS syndrome.

It is noted that the heteroplasmic mtDNA mutation levels increase during passages of cell expansion and undergo a bimodal segregation in the process of reprogramming into iPSC and neuronal differentiation [25]. However, the maintenance of stable heteroplasmy levels is crucial for determining the consequent association with the phenotype. In present studies, we determined the heteroplasmy levels of m.A3243G mutation in both the parental MELAS-fibroblasts and the fibroblast-derived MELAS-iNs. Our results showed that the heteroplasmy levels in the MELAS iNs were compatible with that of the parental MELAS fibroblasts and that both cell clones remained at stable heteroplasmic levels during the experiments, suggesting the iNs as a stable platform for mtDNA-mutation associated studies. Following these findings, we then asked whether the heteroplasmic levels link with the expression of RC complexes, given that the m.A3243G mutation causes mt-tRNA translational defects resulting in a deficiency of RC complexes I and IV. As expected, the expressions of complexes I and IV were reduced profoundly in the MELAS-iNs and parental fibroblasts harboring high heteroplasmy, decreased remarkably in the MELAS iNs and parental fibroblasts harboring intermediate heteroplasmy, and were compatible with that of control in the MELAS iNs and parental fibroblasts harboring low heteroplasmy. The decrease in the RC complex’s expression is parallel with the heteroplasmy in both the MELAS iNs and fibroblasts and the RC complexes deficiency is apparent when a certain threshold of heteroplasmy is reached. Furthermore, the current results, together with our previous studies with MELAS fibroblasts and MELAS iPSC, demonstrated a discrepancy in the RC complex’s deficiency between the MELAS fibroblasts, iPSC, and iNs harboring equivalent heteroplasmy [25,31]. These findings indicate that mtDNA maintenance, transcript, and expression vary in different tissues leading to the tissue-specific manifestation of mitochondrial diseases [32]. Furthermore, the nDNA-encoded proteins involved in mt-tRNA maturation modify the expression of mtDNA mutations in the mt-tRNA genes, leading to the heterogeneity of mitochondrial pathophysiologies [33].

Under normal physiological conditions, the majority of endogenous ROS is produced in mitochondria during the generation of energy through the electron transport chain of complexes I and III [34]. While under pathological conditions, an imbalance between mitochondrial ROS production and removal leads to the elevation in ROS levels, triggering further ROS formation, which causes perceptible mitochondria and cell injury underlying the pathogenesis of mitochondrial diseases [34]. Several studies have indicated that the defect in complex I leads to the elevation in cellular ROS levels and has been associated with a broad spectrum of mitochondrial diseases, such as MELAS syndrome and Leigh syndrome, and neurodegenerative pathologies [34]. Given that the MELAS-iNs showed complexes I and IV deficiency, an increase in ROS production in these cells is to be expected. In agreement with these findings, significant elevation in the ROS levels was observed in the MELAS-iNs harboring high heteroplasmy concomitant with a profound deficiency oin complex I. Notably, MELAS-iNs harboring intermediate heteroplasmy had ROS levels comparable to that of the control, although these neuronal cells showed 87% reduction in complex I. In contrast to our MELAS-iNs, previous studies with MELAS-fibroblasts harboring low (30% and 43%, respectively) m.A3243G heteroplasmy demonstrated a significant increase in ROS levels [35,36]. Glioblastoma cybrids harboring 80% m.A3243G heteroplasmy had ROS levels compatible with that of the control cybrids [11]. These findings indicate that the impact of m.A3243G heteroplasmy on the production and removal of intracellular ROS levels is heterogeneous between tissues and that the physiological levels of ROS in different tissues highly depends on the energy loads to meet the cellular response [34]. Moreover, the nuclear genetic factors and mitochondrial haplogroups modify the production of ROS and the adaptive response to counteract oxidative stress, leading to a heterogeneous threshold between individuals with same heteroplasmy in eliciting the pathological phenotype [37,38].

It has been noted that fulminant elevation in ROS induces a prolonged opening of mitochondrial permeability transition pores, leading to a simultaneous disruption of ΔΨm [34]. Although it was noted that the m.A3243G mutation causes an increase in ROS levels and a decrease in ΔΨm in mutant fibroblasts [35,36], its impact on neuronal cells to elicit mitochondrial depolarization has not been elucidated yet. In the current study, we observed a significant reduction in ΔΨm in those MELAS-iNs harboring high m.A3243G heteroplasmy. Furthermore, those MELAS-iNs containing higher ROS levels showed more reduction in ΔΨm. This finding is in agreement with previous studies relating to complex I-deficient patient-derived fibroblasts in that there is a significant inverse correlation between ΔΨm and cellular ROS levels [39]. Additionally, the MELAS-iNs bearing an intermediate m.A3243G heteroplasmy concomitant with an 87% reduction in complex I activity showed ROS levels and ΔΨm comparable to that of the control. Previous studies with MELAS-fibroblasts harboring 43% m.A3243G heteroplasmy concomitant with a 65% reduction in complex I activity revealed a dissipation in ΔΨm [35]. These results suggest the threshold of m.A3243G heteroplasmy and levels of complex I activity required to elicit mitochondrial dysfunction varies between tissues. Additionally, the adaptive response to compensate for the imbalance of the redox environment is heterogeneous in different tissues and in different parts of the same tissues [34].

It has been noted that the deficiency of RC complexes causes an elevation in ROS, a decrease in ΔΨm, and a reduction in bioenergetics. However, the association of m.A3243G mutation and impaired bioenergetics was demonstrated in limited studies using MELAS patient-derived fibroblasts and iPS cells harboring a high heteroplasmic mutation load [25,31,36]. Moreover, studies related to the impact of m.A3243G mutation on mitochondrial respiratory function in patient-derived neuronal cells is limited to a report of MELAS-derived induced neurons carrying 75% heteroplasmy, a report of MELAS-iPSC derived neurons cells bearing 65% heteroplasmy, and a report of MELAS-iPSC-derived neural progenitor cells bearing 95% heteroplasmy [18,40,41]. Nonetheless, these previous studies did not document the influence of various levels of m.A3243G heteroplasmy on the bioenergetic status among patient-derived neuronal cells. In the current study, we first reported the correlation between the mitochondrial respiratory profiles and m.A3243G heteroplasmy ranging from high (>95%), intermediate (68%), to low (20%) levels in the MELAS-iNs. The bioenergetic parameters including basal respiration, ATP-linked respiration, maximal respiration, and spare respiratory capacity were significantly decreased in the MELAS-iNs harboring the high and intermediate heteroplasmy of the m.A3243G mutation. Notably, the MELAS-iNs carrying a low m.A3243G heteroplasmy had mitochondrial respiration comparable to that of the control, while the MELAS-iNs harboring high m.A3243G mutation showed a most remarkable reduction in respiratory profiles. Moreover, the reduction in bioenergetics is parallel with the reduction in the RC complex’s activity in the MELAS-iNs, confirming the close link between m.A3243G heteroplasmy levels, complex’s activity, and mitochondrial dysfunction.

Dynamic changes in mitochondrial morphology are frequently observed in cells responding to the alternation of bioenergetic status. It has been shown that the inhibition of complex I, II, III, or V causes mitochondrial fragmentations and cell death [42,43]. In previous studies, MELAS-fibroblasts and MELAS-iPS cells harboring more than 80% heteroplasmy in the m.A3243G mutation showed a reduction in bioenergetic function as well as mitochondrial fragmentation [25,44]. However, the influence of various levels of m.A3243G heteroplasmy on mitochondrial morphology in neurons has not been documented. Herein, we quantitatively measured the mitochondrial size and shape as well as the overall connectivity and morphological complexity of the mitochondrial network to unveil the formation of fragmented and filamentous mitochondria in the MELAS-iNs. In parallel with the reduction in bioenergetics, the MELAS-iNs bearing high and intermediate heteroplasmy showed characteristics of fragmented mitochondria through the significant reduction in mitochondrial area, perimeter, aspect ratio, and form factor as well as mitochondrial connection and branching degree compared to the filamentous mitochondria in the control-iNs. This finding agrees with previous studies concerning MELAS-skeletal muscles and MELAS-cybrids in that mitochondrial dysfunction resulting from pathogenic mtDNA variants leads to mitochondrial fragmentation [45]. In this study, a decrease in OXPHOS capacity in the MELAS-iNs resulted in an increase in ROS levels, dissipation of ΔΨm, reduction in bioenergetics, and mitochondrial fragmentation. Of note, both the MELAS-iNs bearing low heteroplasmy and its counterpart control-iNs were derived from aged individuals and revealed mitochondrial fragmentation concurrent with a moderate decrease in basal and ATP production respiration. It has been suggested that the fission of mitochondria acts as an adaptive mechanism to mitigate bioenergetic insufficiency during aging [46]. Overall, our studies indicate the shift of mitochondrial dynamics toward fission and fragmentation in MELAS neurons harboring high and intermediate heteroplasmy and neurons derived from aged individuals. It has been noted that in the fragmentation status, dysfunctional mitochondria are segregated from the functional mitochondrial network to reduce further mtDNA damage via oxidative stress derived from the damaged mitochondria [45]. Several studies have documented the parallel changes in oxidative stress and mitochondrial dynamics. It has shown that patient-derived fibroblasts with severe complex I deficiency showed a prominent increase in ROS levels and fragmented mitochondria, while fibroblasts with moderate deficiency of complex I activity displayed a moderate increase in ROS levels concurrent with normal mitochondrial morphology [47]. The mechanism to regulate mitochondrial dynamics in response to mitochondrial dysfunction has been elucidated in several studies. In a Drosophila model for wound healing, high levels of ROS triggers Drp1 (dynamin-related protein 1)-mediated fragmentation of mitochondria, while low levels of ROS promotes fusion and network formation in mitochondria [48]. In keeping with this note, previous studies with MELAS-fibroblasts harboring 82% m.A3243G heteroplasmy demonstrated elevation in Drp1 expression concurrent with highly fragmentated mitochondria [44]. Moreover, it has been noted that elevated ROS levels trigger the depolarization of ΔΨm and mitochondrial fragmentation, suggesting the participation of ROS levels and ΔΨm in modeling the mitochondrial dynamics [49]. Studies in mammalian cells further indicated that mitochondrial fragmentation is stimulated through the activation of the proteolytic cleavage of OPA1 (optic atrophy-1), an essential factor for mitochondrial fusion and the maintenance of inner membrane structure, in a ΔΨm-dependent manner [50]. Consistent with this note, previous studies with MELAS-muscle fibers revealed that the dissipation in ΔΨm induces the proteolytic processing of OPA1 concomitant with the fragmentation of mitochondria [45]. Collectively, all these notes indicated that the activation of Drp1 via ROS and the downregulation of OPA1 via the dissipated ΔΨm contribute to the shift of mitochondrial dynamics toward fragmentation in MELAS cells. Nonetheless, the inhibition of mitochondrial fragmentation promotes an excessive increase in intracellular ROS levels and cell death in MELAS-fibroblasts [44]. Mitochondrial transplantation recovers bioenergetics and mitochondrial morphology in osteosarcoma MELAS-cybrids and treatment with cell-permeable exogenous antioxidant restores mitochondrial network in rhabdomyosarcoma cybrids harboring m.A3243G mutation [51,52]. Accordingly, these findings suggested that sequestration of the dysfunctional mitochondrial from the healthy mitochondria through mitochondrial fission could be a cytoprotective strategy to reduce cellular cytotoxicity in MELAS cells. Furthermore, the genetic and/or pharmacological modulation of bioenergetics and oxidative stress provide promising therapeutic approaches to restore mitochondrial dynamic balance in MELAS syndrome and mitochondrial diseases.

## 5. Conclusions

In summary, our studies showed that the iNs, through the direct reprogramming of the MELAS patient-derived fibroblasts, exhibited the neuronal phenotype, retained the status of m.A3243G heteroplasmy concurrent with the deficiency of RC complexes, and adopted the donor age-dependent phenotype. We first demonstrated the impact of various levels of m.A3243G heteroplasmy on the expression of RC complexes, neuronal ROS levels, ΔΨm, bioenergetic profiles, and mitochondrial morphology and network in neurons. High and intermediate levels of m.A3243G heteroplasmy in neurons resulted in an impairment of RC complexes, reduction in mitochondrial respiration, and fragmentation of mitochondrion. The impairment of bioenergetics and the imbalance of mitochondrial dynamics contribute to the pathomechanism of the neurological phenotype of MELAS syndrome and the concurrent elevation in ROS levels, and the disruption of ΔΨm contributes to the initiation of an early onset neurological manifestation. Our studies validated iNs as a reliable platform for studies in the neuronal aspects of neurodegenerative disorders and mitochondrial diseases. These results help us in understanding the impact of various heteroplasmic levels on mitochondrial bioenergetics and dynamics in neurons and in underlying the pathomechanism of the neurological manifestations of MELAS syndrome. Furthermore, these findings provide targets for further pharmacological approaches concerning mitochondrial diseases.

## Data Availability

The datasets used and/or analyzed during the current study available from the corresponding author on reasonable request.
